# Editorial: Silent Seizures and Memory Loss in Alzheimer's Disease

**DOI:** 10.3389/fneur.2021.648650

**Published:** 2021-03-08

**Authors:** Keith Vossel, Elissaios Karageorgiou

**Affiliations:** ^1^Mary S. Easton Center for Alzheimer's Disease Research, Department of Neurology, David Geffen School of Medicine at University of California, Los Angeles, Los Angeles, CA, United States; ^2^Sleep & Memory Center, Neurological Institute of Athens, Athens, Greece

**Keywords:** seizures, epileptiform activity, Alzheimer's disease, dementia with Lewy bodies, electroencephalography

Brain rhythms are the foundation of normal cognition and behavior, yet remarkably little is known about changes in brain network activity that accompany cognitive decline in diseases of aging. In particular, seizures in dementia are important to recognize due to their harmful impact on patients and their potential to respond to existing therapy. Scientific models of Alzheimer's disease (AD) and human observational studies show strong correlations between seizures in AD and cognitive decline, yet many questions remain: (1) what cognitive functions are most impacted by seizures and epileptiform activity in AD; (2) what are the best methods to detect seizures, epileptiform activity, and related network dysrhythmias in AD; (3) how do late-onset epilepsy and subclinical epileptiform activity relate to AD biomarkers in patients with mild cognitive impairment; and (4) what is the impact of seizures on cognition in non-AD disorders?

In this Research Topic, seven manuscripts from academic institutions in England, Finland, Hungary, Italy, and the United States begin to address these questions. These manuscripts cover a broad array of methods including electroencephalography (EEG) and polysomnographic recordings in transgenic models of AD and dementia with Lewy bodies, scalp and foramen ovale EEG recordings in humans, and longitudinal clinical assessments. These studies advance our understanding of network dysrhythmias in AD and related disorders and enhance our capability to develop and test therapies to normalize brain rhythms in dementia. We present these investigations in three categories: (1) preclinical studies in transgenic mouse models, (2) human EEG recording and analyses, and (3) human biomarkers and longitudinal studies.

## Preclinical Studies

Transgenic mouse models overexpress human disease proteins harboring familial mutations linked to dementia and simulate important aspects of neuropathology and cognitive deficits in dementia. EEG recordings in these models are useful to demonstrate effects of disease protein expression on broad network activity. EEG electrodes in mice cover many cortical and subcortical brain regions and have higher precision and sensitivity for detecting network abnormalities compared to human scalp electrode recordings.

Gureviciene et al. sought to identify EEG features related to amyloid-β brain pathology, their association with seizures, and their potential attenuation through antiseizure drug use. Specifically, they studied the surface and depth electroencephalographic features of transgenic mice with APP/PS1 mutations and compared them to wild-type mice, identifying eight distinct epileptiform patterns derived through specific classification criteria across all mice. Four of these patterns (pure cortical spikes, slow-wave discharges, cortical-hippocampal spikes with hyperpolarization, and giant spikes) were more common in transgenic mice. Of these, giant spikes alone had a strong association with seizure occurrence and were followed by hyperpolarization, that might reflect aspects of temporal slowing also observed in older adults with epileptiform activity (see Babiloni et al. in this Research Topic). These spikes also occurred almost exclusively during sleep, in keeping with what is observed in humans with AD (1–3). Of further clinical significance, giant spikes and cortical-hippocampal spikes responded to treatment with the antiseizure drugs levetiracetam and ethosuximide, supporting the potential effectiveness of these drugs in humans with AD and seizures or subclinical epileptiform activity.

Patients with α-synuclein disorders, including Parkinson's disease and Lewy body dementias, are also at increased risk for seizures, as well as cortical myoclonus (4, 5). Peters et al. showed a link between network hyperexcitability and cognitive decline in a model of α-synucleinopathy. The investigators performed cortical EEG recordings in transgenic mice expressing human α-synuclein with the A53T mutation linked to familial Parkinson's disease. This study found epileptiform discharges, occurring mostly during non-REM sleep, as well as epileptic myoclonus and EEG slowing in transgenic mice. Seizures and epileptiform activity in A53T mice precede, and likely contribute to, inhibitory remodeling and synaptic and cognitive impairment. The seizure and cognitive phenotype in A53T mice depends on endogenous tau and involves post-synaptic mechanisms (6, 7). This study adds to the understanding of aberrant network activity due to α-synuclein pathology and supports future translational approaches, such as anti-seizure drugs and tau reduction, for α-synuclein disorders.

## Human EEG Studies

Scientists are striving to gain better sensitivity to detect seizures and epileptiform activity that occur in the mesial temporal lobes (mTL), which are the most common regions affected in AD. Seizures within these regions are often undetectable with scalp EEG recordings. Lam et al. present two approaches to identify mTL seizures in AD: foramen ovale recordings and algorithms to infer mesial temporal lobe activity from scalp EEG recordings. The authors discuss how foramen ovale recordings can be critical to precisely diagnose and treat silent mesial temporal lobe seizures in AD. This approach has many advantages over empirical treatment but is not often feasible. To address this limitation, the authors are devising algorithms using combined FO and scalp recordings to determine signature features on scalp EEG associated with silent mTL seizures. Such approaches will be very useful to broadly study silent mesial temporal lobe seizures in AD and for large-scale clinical trials.

Babiloni et al. pursue an alternative approach, wherein they sought to identify surrogate electrophysiologic markers obtained in routine 30-min EEG recordings as predictors of epileptiform activity in people with amnestic cognitive impairment of non-AD pathology and without a diagnosis of epilepsy or seizures. Epileptiform activity in the form of paroxysmal spikes, sharps, slow-wave discharges, and giant spikes was identified in 41% of patients. The presence of epileptiform activity occurred in people who had quantifiably more delta slowing over the temporal lobes, in keeping with animal studies in AD (see Gureviciene et al. in this Research Topic). The implications, in addition to suggesting a neurophysiologic connection between epileptiform activity and temporal slowing, also support the clinical utility of temporal slowing in pursuing detailed assessments for epileptiform discharges in people with amnestic cognitive impairment and temporal slowing, irrespective of the underlying pathology.

## Biomarkers and Longitudinal Studies

Cases presenting with new-onset seizures of unknown etiology and cognitive symptoms are complex and require new tools for precise workup and management. Having biomarkers and clinical features that distinguish degenerative from non-degenerative cases and epileptic from non-epileptic presentations are urgently needed.

Cesarini et al. made the intriguing discovery that patients with late-onset epilepsy of unknown etiology and mild cognitive impairment (MCI) have lower amyloid-β levels in cerebrospinal fluid, indicating that the seizures in these patients, or a large subset, could be a consequence of early Alzheimer's pathology. These findings signify that Alzheimer's biomarkers could be useful for proper management in patients with MCI and new-onset seizures. They also found that patients with MCI and epilepsy are worse in global cognitive measures, visuo-spatial abilities, and executive functions compared to MCI patients without epilepsy, indicating non-amnestic features associated with epilepsy in MCI.

Horvath et al. similarly found that patients with mild AD and seizures have more visuo-spatial dysfunction and greater parietal cortex atrophy compared to those without seizures or subclinical epileptiform activity. They found epileptiform activity in 52% (14 of 27) of patients with mild AD using 24-h EEG recordings. Seven of those with epileptiform activity had overt seizures. These findings add to evidence of seizures and epileptiform activity in early AD and indicate that early parietal lobe dysfunction is associated with epilepsy in AD.

Baker et al. extend on the findings of Horvath et al. by pursuing longitudinal cognitive assessments of people with AD with and without a seizure history. This study is unique because it is one of the first to follow these AD subgroups longitudinally, and few patients were treated with antiseizure drugs. They identified 102 people with AD from the Presentation of Epileptic Seizures in Dementia (PrESIDE) study and divided them according to the presence (*N* = 29) or absence (*N* = 73) of an epilepsy history. Although patients with epilepsy did not significantly differ from patients without epilepsy on baseline cognitive function, their daily functioning was worse per informant-completed questionnaires. When repeating cognitive testing after a year in 27 patients with seizure history and 45 patients without seizures, patients with a seizure history had faster cognitive decline and their daily functioning remained worse. This decline was especially evident in executive functions of attention and fluency, which is notable because decline in executive function was also observed in AD patients exhibiting subclinical epileptiform activity (1).

## Summary

This Research Topic provides new methodologies to detect seizures and epileptiform activity in mouse models and humans and demonstrates cognitive impairments associated with seizures that could respond to antiseizure drugs. A common theme is that a newly described variant of AD, the epileptic variant, is emerging and deserves careful consideration due to differences in age-at-onset, phenotype, progression, and treatment. The epileptic variant of AD could explain the wide variability in AD presentation and progression and should be accounted for in future clinical trials. Its contribution is not trivial when considering that up to 60% of patients with AD have seizures and subclinical epileptiform activity (1, 8, 9). Further investigations into late-onset epilepsy in related disorders, such as dementia with Lewy bodies and non-degenerative amnestic cognitive impairment, are also warranted. Studies to date have been limited by small sample sizes and lack of participant diversity illustrating the great need for multi-center studies and more inclusive recruitment in future investigations.

## Author Contributions

KV and EK drafted and revised the manuscript. Both authors contributed to the article and approved the submitted version.

## Conflict of Interest

The authors declare that the research was conducted in the absence of any commercial or financial relationships that could be construed as a potential conflict of interest.
